# An Annotated List of Legume-Infecting Viruses in the Light of Metagenomics

**DOI:** 10.3390/plants10071413

**Published:** 2021-07-10

**Authors:** Elisavet K. Chatzivassiliou

**Affiliations:** Plant Pathology Laboratory, Department of Crop Science, School of Plant Sciences, Agricultural University of Athens, 11855 Athens, Greece; echatz@aua.gr

**Keywords:** cool season legumes, forage legumes, grain legumes, insect-transmitted viruses, pulses, integrated control, seed-transmitted viruses, virus epidemiology, warm season legumes

## Abstract

Legumes, one of the most important sources of human food and animal feed, are known to be susceptible to a plethora of plant viruses. Many of these viruses cause diseases which severely impact legume production worldwide. The causal agents of some important virus-like diseases remain unknown. In recent years, high-throughput sequencing technologies have enabled us to identify many new viruses in various crops, including legumes. This review aims to present an updated list of legume-infecting viruses. Until 2020, a total of 168 plant viruses belonging to 39 genera and 16 families, officially recognized by the International Committee on Taxonomy of Viruses (ICTV), were reported to naturally infect common bean, cowpea, chickpea, faba-bean, groundnut, lentil, peas, alfalfa, clovers, and/or annual medics. Several novel legume viruses are still pending approval by ICTV. The epidemiology of many of the legume viruses are of specific interest due to their seed-transmission and their dynamic spread by insect-vectors. In this review, major aspects of legume virus epidemiology and integrated control approaches are also summarized.

## 1. Introduction

Legume is a term used for the plant or the fruit/seed of plants belonging to the Fabaceae (Leguminosae) family. Pulses or grain legumes include species the seeds of which are used as a protein source for humans and animals, and in oil production, while forage legumes are sown in pastures and are used for silage and forage. Furthermore, legumes are known to improve the fertility potential of the soil, therefore they may constitute major components for the sustainable management of farming systems [1].

Cultivated legumes are known to be susceptible to natural infection by a plethora of viruses. Several comprehensive publications have reviewed the viruses affecting specific legume crops or production areas (i.e., [2,3,4,5,6,7]). However, the number of legume viruses identified continues to increase in the light of ongoing research, while in the era of metagenomics analysis, the application of new molecular technologies and high-throughput sequencing (HTS) technologies contribute highly to the discovery of new viruses [8,9]. Some of these novel viruses are officially classified, while others are still missing some critical features for their final taxonomic assignment.

This review aims to present an updated list, though non-exhaustive, of the viruses found to naturally infect legumes. Species taken into consideration were the most important annual cool season legumes, including chickpea or garbanzo bean (*Cicer arietinum* L.), faba or broad bean (*Vicia faba* L.), lentil (*Lens culinaris* Medik.), field pea (*Pisum sativum* L.), and the warm season ones, common bean (*Phaseolus vulgaris* L.), cowpea (*Vigna unguiculata* (L.) Walp.), and groundnut or peanut (*Arachis hypogaea* L.). The most important forage legumes considered were the perennial alfalfa or lucerne (*Medicago sativa* L.), clovers (*Trifolium* spp.), and annual medics (*Medicago* spp.).

The updated list of these legume-infecting viruses includes 168 viruses, which belong to 39 genera and 16 families (Table 1). The species listed are those recognized by the International Committee on Taxonomy of Viruses, until 2020 [10]. To accommodate the genetic divergence in the virosphere, the ICTV has recently adopted a 15-rank classification hierarchy of viruses to closely align with the Linnaean taxonomic system [11] however, here only the family-level classification of the legume-infecting viruses is presented.

## 2. Viruses Infecting Major Legumes

### 2.1. Alfalfa (Lucerne)

At least 36 pathogenic viruses, belonging to 24 genera of 11 families, have been reported to naturally infect alfalfa crops (Table 1) [14,15,16,17,18,19]. Alfalfa mosaic virus (AMV) is the most widespread virus that highly impacts alfalfa yield worldwide [14]. The recently discovered alfalfa leaf curl virus (ALCV) is also causing a severe disease of global distribution and impact [20]. Cucumber mosaic virus (CMV), lucerne transient streak virus (LTSV), lucerne Australian latent virus (LALV), bean leafroll virus (BLRV), beet western yellows virus (BWYV), soybean dwarf virus (SbDV), and turnip yellows virus (TYV) are also of potential impact for alfalfa [14,16,17]. Their widespread infections, although in some areas considered of minor importance, might still contribute to diseases of unknown impact [16].

The AMV-infected alfalfa plants show symptoms of different severity, from symptomless infections (especially under warm conditions) to mottle or light green vein banding and rings, accompanied by leaf deformation and plant stunting. ALCV causes plant stunting, leaf curling, crumpling, and shriveling. CMV infections cause light-green mosaic or mottle on slightly malformed leaves. LTSV causes transient yellow streaks or flecks along the leaf veins, while BLRV induces mild, transient yellowing of older leaves. The LALV infections of alfalfa remain latent. Alfalfa also exhibits a number of viral symptoms of still unknown etiology [16].

The list of alfalfa viruses is continuously increasing, and thanks to the use of HTS when exploiting some alfalfa disease syndromes, the identification of numerous novel viruses, such as ALCV, alfalfa dwarf virus, alfalfa enamovirus 1, alfalfa virus S, and alfalfa virus F, was made possible, and alfalfa was also confirmed as a new host for known viruses, such as cactus virus X, chickpea chlorosis Australia virus (CpCAV), strawberry latent ringspot virus (SLRSV), and cycas necrotic stunt virus [15,21,22,23,24]. Additionally, a number of new, but not yet approved, viruses have been identified, namely alfalfa-associated nucleorhabdovirus (AaNV; genus *Nucleorhabdovirus*), alfalfa ringspot-associated virus (ARaV; genus *Emaravirus*), cnidium vein yellowing virus (CnVYV; Unassigned), lychnis mottle virus (LycMoV; Unassigned), medicago sativa marafivirus 1 (MsMV1, genus *Marafivirus*), and Zhuye pepper nepovirus (ZPNu; genus *Nepovirus*) [21,22,23,25].

### 2.2. Clovers and Annual Medics

Thirty-two viruses (15 genera, 10 families) have been reported to infect clovers (*Trifolium* spp.) (Table 1) [14,26,27,28,29,30]. Red clover nepovirus A (RCNVA, genus *Nepovirus*), red clover-associated varicosavirus (*Rhabdoviridae* family), and phasey bean mild yellows virus (PBMYV, genus *Polerovirus*) are suggested as new clover pathogens still pending official classification [31,32,33].

Annual medics (*Medicago* spp) have been reported to be infected by 10 viruses, from seven genera and six families (Table 1) [26,34,35,36].

### 2.3. Common Bean

Globally, beans are reported to be infected naturally by at least 83 viruses belonging to 24 genera of 12 families (Table 1) [4,5,37,38,39,40,41,42,43,44,45,46,47,48,49,50,51,52,53,54,55,56,57,58,59,60,61,62,63,64,65]. Among these viruses the potyvirids bean common mosaic virus (BCMV) and bean common mosaic necrosis virus (BCMNV) are considered the most important ones. However, an increasing number of begomoviruses, such as bean golden mosaic virus (BGMV), bean golden yellow mosaic virus (BGYMV), bean dwarf mosaic virus (BDMV), bean summer death virus, bean yellow dwarf virus, and bean calico mosaic virus (BCaMV), are emerging in the tropics, causing highly destructive diseases. Other major bean viruses are AMV, CMV, bean yellow mosaic virus (BYMV), and cowpea chlorotic mottle virus (CCMV) [4,5,39,41].

BCMV and BCMNV often cause common mosaic associated with leaf curling and malformations, with general stunting of the plant. BCMNV may cause (in varieties possessing the dominant I resistant gene) the lethal “black root” disease which is characterized by red-brown expanding spots on leaves that become necrotic. Infections by begomoviruses are mostly associated with yellow mosaic diseases. BYMV-infected beans show crinkling, yellow mottling, and necrotic areas along the veins of infected leaves. AMV infections are associated with mosaic or mottle, while CMV strains can induce mild mosaic to severe plant malformation. CCMV is the causal agent of “bean yellow stipple disease” [4,5,7,39,41,66].

Recently, sequences of the Ethiopian tobacco bushy top virus were retrieved from common bean using HTS [67]. Common bean has been identified as a host of Pelargonium vein banding virus (PVBV, genus *Badnavirus*, family *Caulimoviridae*), which is still pending official classification [59].

### 2.4. Cowpea

Cowpea is susceptible to at least 33 legume viruses from 16 genera and 11 families (Table 1) [5,68,69,70,71,72,73]. The highly damaging viruses which are detected in most of the cowpea-producing countries include the seed-borne blackeye cowpea mosaic strain (BCMV), cowpea aphid-borne mosaic virus (CABMV), cowpea mosaic virus (CPMV), cowpea severe mosaic virus (CSMV), cowpea mottle virus (CPMoV), CMV, CCMV, Southern bean mosaic virus (SBMV), and the non-seed-borne CCMV, and cowpea golden mosaic virus (CGMV) [5,73].

The potyvirids CABMV and BCMV-blackeye cowpea mosaic strain produce on susceptible cowpeas an indistinguishable veinal and interveinal chlorosis, or dark-green vein banding. CPCMV, CPGMV CPMV, CSMV, and CMV cause different mosaic symptoms. SBMV may induce from symptomless infections to severe mottle or mosaic with leaf deformations [5,7,73,74].

Recently-discovered cowpea-infecting viruses that are pending official classification are cowpea polerovirus 1 (CPPV1), cowpea polerovirus 2 (CPPV2), and possibly two tombusviruses [75,76].

### 2.5. Chickpea

Chickpeas are reported to be naturally infected with at least 40 viruses, from 19 genera and 11 families worldwide (Table 1) [3,5,7,33,77,78,79,80,81,82,83,84]. Globally, viruses of major economic importance are the ones causing or being associated with chickpea stunt and chlorotic dwarf diseases, including: (i) the aphid-transmitted BLRV, BWYV, chickpea chlorotic stunt virus (CpCSV), cotton leaf roll dwarf virus (CLRDV), cucurbit aphid-borne yellows virus (CABYV), faba bean necrotic yellows virus (FBNYV), faba bean yellow leaf virus (FBYLV), faba bean necrotic stunt virus (FBNSV), pea necrotic yellow dwarf virus (PNYDV) and SbDV; and (ii) the leafhopper-transmitted CpCAV, chickpea chlorotic dwarf virus (CpCDV), chickpea yellow dwarf virus (CpYDV), chickpea chlorosis virus (CpCV), chickpea redleaf virus (CpRLV), and chickpea yellows virus (CpYV). The aphid- and seed-transmitted AMV, BYMV, CMV, and pea seed-borne virus (PSbMV), although present worldwide, are considered of minor importance [5,6,82].

The main viral disease affecting chickpea is the “stunt disease” with symptoms of stunting, leaf rolling, yellowing (or reddening), premature necrosis and, if the plants survive, poor pod-set. Poor pod-set was initially attributed only to BLRV and FBNYV, but recently it has been associated with infections by: the poleroviruses BLRV, BWYV, CpCSV, CLRDV, and CABYV; the nanoviruses FBNYV, FBYLV, FBNSV, MDV, PNYDV, and SCSV; the luteovirus SbDV; or the mastreviruses CpCDV, CpYDV, CpCV, CpRLV, and CpYV. AMV, BYMV, CMV, and PSbMV are associated with mosaic and mottle symptoms [5,6,7,82].

Deep sequencing approaches identified chickpea as a new host of tomato mottle mosaic virus and of hop stunt viroid (HSVd, genus *Hostuviroid*, family *Pospiviroidae*) [81]. Chickpea was also found infected with the polerovirus PBMYV [33], that is pending official classification.

### 2.6. Faba Bean

Worldwide, faba bean is naturally infected by at least 40 viruses from 20 genera and 11 families (Table 1) [3,4,5,17,33,84,85,86,87,88,89,90,91]. The aphid-transmitted FBNYV, and possibly the related FBYLV, FBNSV, PNYDV, subterranean clover stunt virus (SCSV), and milk vetch dwarf virus (MVDV), are considered as the most important virus(es) for faba bean, which may cause complete crop loss [6]. Globally, BLRV, and possibly the related SbDV, BWYV, CpCSV, CABYV, and CpCDV, may have also significant impact. Bean yellow mosaic virus and broad bean mottle virus are also listed as important faba bean viruses [4,5,6,84,89].

FBNYV and related viruses cause necrotic yellow symptoms in faba bean plants, stunting, and poorly developed new shoots, leaves, and flowers. Leaves are small, rolled, and present interveinal chlorosis, which develops to necrosis. Infections might result in the death of the plants. Infection with BLRV (or related viruses) causes a leaf roll disease characterized by interveinal chlorosis, yellowing, stunting, leaf rolling, reddening and thickening of the leaves, suppression of flowering, and pod setting; infections before blooming may result in compete yield loss. BYMV is associated with mosaic and necrosis and BBSV with mottling, marbling, diffuse mosaic, and often with leaf malformation and plant stunting [4,5,7,84,89].

Except viruses, citrus exocortis viroid (CEVd, genus *Pospiviroid*, family: *Pospiviroidae*) [92] and the non-officially classified polerovirus PBMYV [33] have been reported to naturally infect faba bean plants.

### 2.7. Groundnut

Worldwide, around 27 viruses from 11 genera and 10 families have been reported to naturally infect groundnut. Groundnut bud necrosis orthotospovirus (GBNV), tomato spotted wilt orthotospovirus (TSWV), Indian peanut clump virus (IPCV), peanut clump virus (PCV), peanut mottle virus (PeMoV), and BCMV (synonym peanut stripe virus), tobacco streak virus (TSV), CMV, and the symbiotic association of groundnut rosette virus (GRV) with groundnut rosette assistor virus (GRAV) are considered the most economically important ones. Groundnut rosette (chlorotic, green, or mosaic) disease is caused by the complex of GRAV, GRV, and a satellite RNA. Often IPCV and PCV infections can be confused with green rosette. TSWV induces spotted wilt and GBNV bud necrosis disease, which are similar in appearance. TSV-infected groundnut plants show stem necrosis. PeMoV and PStV infections are associated with mottle and stripe, and CMV infections with yellow mosaic [5,93,94,95,96].

### 2.8. Field Pea

Worldwide, peas can be naturally infected with at least 42 viruses from 18 genera and 11 families (Table 1) [3,4,5,33,55,97,98,99,100,101,102]. The most widespread and important ones are AMV, BLRV, BYMV, BWYV, FBNYV, pea early browning virus (PEBV), pea enation mosaic virus (the symbiotic association of PEMV-1, and PEMV-2), PNYDV, PSbMV, pea streak virus (PSV), TuYV, and red clover vein mosaic virus (RCVMV) [4,5,6,99,102].

AMV-infected pea plants exhibit curling, chlorosis, and sometimes necrotic lesions on leaves, streaks on the stem, malformations, and general stunting. BLRV symptoms include chlorosis, upward curling of leaves (top yellows), and stunting. BYMV and PSbMV infections are mostly associated with mosaic and veinal chlorosis symptoms. PSbMV-infected plants may also show severe stunting, especially in seed-borne infections. PEMV-infected peas exhibit mosaic, leaves show chlorotic and translucent spots, vein clearing, downward curling (at the early stages of infection), growth deformations (epinasty, stunting, rugosity, loss of apical dominance), and enations (hyperplastic outgrowths) associated with the veins underneath the leaf lamina, while pods may be distorted with wards (at later stage of infection). PSV causes brown necrotic streaks on stems and petioles, necrotic lesions on leaves, and wilting of the plant. BWYV and TuYV cause yellowing, rolling, thickening of leaves, and stunting of plants. PNYDV-infected peas show severe dwarfing of plants, yellowing, and leaf-rolling. PEBV causes purplish-brown necrotic discoloration and plant necrosis. The RCVMV-infected pea plants exhibit severe stunting, rosetting of leaves, and plant necrosis in early stages of infection [4,5,7,103,104].

Recent efforts to explore the pea virome identified pea-associated emaravirus (PaEV) (*Fimoviridae* family) and PBMYV (genus *Polerovirus*) [33,102] which are not officially approved yet.

### 2.9. Lentil

About 24 viruses from 16 genera and 9 families have been reported to infect lentil in nature (Table 1). The aphid-transmitted BLRV, BWYV, CpCSV, FBNYV, SbDV, and the leafhopper-transmitted CpCDV are associated with severe lentil diseases. BYMV, broad bean stain virus (BBSV), CMV, PEMV, and PSbMV are also considered important lentil viruses [2,3,5,6,82,84]. BLRV, BWYV, CpCSV, FBNYV, and SbDV in lentil cause leaf rolling, reddening, overall yellowing, and stunting symptoms. PSbMV, CMV, PEMV, PeSV, and BYMV cause mosaic and mottling, which is often confused with abiotic diseases (e.g., nutrient deficiencies, physiological disorders, herbicide damage, and waterlogging stress) [2,5,7,82,84]. The new but not officially approved polerovirus PBMYV [32] may also infect lentil.

## 3. Epidemiology of Legume-Infecting Viruses

Plant viruses are obligate pathogens, and their survival depends on their dispersal mechanisms. They can often cause destructive epidemics, especially those transmitted by insects [105,106]. The epidemiology of the insect-transmitted viral diseases is complex, where the susceptible host interacts with alternative virus sources via the activity of the insect vectors, thus resulting in a certain level of infection [107]. Therefore, major factors that affect virus spread and the resulting impact are the susceptibility of the crop, the vector involved, the type of transmission, and the number and type (annual/perennial) of its host plants that may act as alternate viral sources, all of which are affected by the environment [108,109,110].

### 3.1. Types of Insect-Mediated Virus Transmission

The insect-transmitted viruses are characterized by specific interactions with their vectors during transmission. Depending on the time the vector remains viruliferous, they are classified as non-persistent (with short retention time), semi-persistent (with intermediate retention time) or persistent (with extended retention time). Depending on the site at which the virus is retained or the route of movement of the virus within its vector, viruses are termed as non-circulative (associated with the mouthparts or foregut) or circulative (cross multiple membrane barriers and are transported within the vector’s haemolymph). The non-circulative viruses are either non-persistent or semi-persistent, while among the circulative viruses some replicate in the vector’s tissues (circulative propagative viruses), while others do not (circulative non-propagative viruses) [12,106,111,112,113]. Depending on their transmission biology, legume viruses fall into all these transmission classes (Table 1).

#### 3.1.1. The Non-Persistently Transmitted Legume Viruses

The non-persistent plant viruses are mostly transmitted by aphids. They multiply in the epidermal cells of the plants, and thus they are acquired and inoculated during probing by aphids (feeding of seconds to minutes). Due to the short time needed for their transmission, they are transmitted with low vector specificity, by several aphid species, mostly the ones that visit, while seeking a feeding site, but do not colonize the crop (transient species). These viruses are non-circulative, only being associated with the insect’s mouthparts (termed also as stylet-borne viruses). As a result, they can be transmitted immediately after acquisition (no latent period), but they are lost after aphid moulting. Due to the external root of transmission, the vector remains viruliferous for only short periods of time (minutes), thus they can be only spread over short distances. Therefore, their sources of infection are within the crop or in close proximity. The non-persistent legume aphid-transmitted viruses belong to the genera *Alfamovirus*, Cucumovirus, *Fabavirus*, *Macluravirus*, and *Potyvirus*. Those in the *Carlavirus* genus are also non-persistently transmitted by their aphid or whitefly vectors (Table 1) [11,12,13].

#### 3.1.2. The Semi-Persistently Transmitted Legume Viruses

The semi-persistent viruses concentrate in the plant phloem or surrounding tissues, therefore their insect vectors need long feeding times (hours) for acquisition and transmission. These viruses do not enter the insect’s body, but they are associated with its foregut (termed also foregut-borne) and their retention period extends from hours to days. However, similar to the non-persistent viruses, they do not require a latent period, and they are lost when the vector moults. Due to the extended feeding time needed for transmission, they have a narrower range of vector species that colonize the crop. Semi-persistent transmission may be carried out by aphids, whiteflies, and leafhoppers. The semi-persistent legume viruses belong to the aphid-transmitted genera of *Caulimovirus* (which may also be non- persistently transmitted; bimodal transmission), and the whitefly-transmitted genus *Crinivirus*. The beetle-transmitted *Bromovirus*, and *Comovirus* are also reported to be transmitted in a semi-persistent manner (Table 1) [11,12,13].

#### 3.1.3. The Persistently-Transmitted Legume Viruses

Persistent viruses are mostly phloem-limited, therefore they require long feeding periods (of several hours to days) to be efficiently acquired and transmitted by their insect vectors. As a result, the persistent viruses can only be transmitted by species which colonize the crop. They circulate inside the vector’s body, traversing insect tissue membranes to invade multiple organs and they pass through moult. Therefore, a latent period is needed after acquisition for these viruses to finally reach and invade the salivary glands to be successfully transmitted. Persistent viruses exhibit a high degree of vector specificity and vectors may remain viruliferous for some weeks (the circulative viruses) or for their lifetime (the circulative propagative viruses). As a result, they can be spread over long distances and individual insects can infect many plants. Some propagative viruses may be also transmitted to the vector’s offspring. Among the legume-infecting persistent viruses, circulative transmission can be found in the aphid-transmitted genera of *Capulavirus*, *Enamovirus*, *Luteovirus*, *Nanovirus*, *Polerovirus*, and possibly to the viruses not assigned to a specific genus of the *Luteoviridae* family. Indirectly, the circulative transmission applies to viruses in the genus *Umbravirus* that use an assistor luteovirus for their transmission. In addition, the whitefly-transmitted viruses of the *Begomovirus* genus or the leafhopper-transmitted viruses of the genera *Becurtovirus*, *Curtovirus*, and *Mastrevirus* are also transmitted in a persistent, circulative manner. On the other hand, the circulative propagative legume viruses belong to the thrips-transmitted *Orthotospovirus*, the aphid-transmitted *Cytorhabdovirus*, and the leafhopper-transmitted *Marafivirus* genera (Table 1) [11,12,13].

### 3.2. Other Types of Virus Transmission in Legume Viruses

The eriophyid mite-transmitted viruses of the *Alexivirus* genus are transmitted in a semi-persistent manner. The same applies to the nematode transmitted viruses in the genera of *Cheravirus*, *Nepovirus*, *Tobravirus*, and possibly the non-assigned to a specific genus SLRSV (*Secoviridae* family). The chythrid-transmitted *Alphanecrovirus*, *Dianthovirus*, *Tombusvirus* genera, and pea stem necrosis virus (*Tombusviridae* family), and the plasmophorid-transmitted genera *Pecluvirus* and *Pomovirus*, show characteristics of persistent transmission. In the field, legume viruses in the *Potexvirus*, *Sobemovirus*, *Soymovirus*, *Tombusvirus*, and *Tobamovirus* are readily transmitted by contact, and may be sometimes mediated by insects. Viruses in the *Dianthovirus* and *Tombusvirus* genera may be also readily transmitted via soil without a vector. Viruses in the *Bromovirus*, *Comovirus*, and *Tymovirus* genera are reported to be semi-persistently transmitted by beetles, but their transmission is sometimes considered by contact; this possibly applies also to the unassigned bean mild mosaic virus (*Tombusviridae* family). Viruses in the *Ilarvirus* genus are transmitted by pollen and their transmission may be facilitated by thrips. Finally, the vector(s) and transmission type of the legume viruses belonging to the genus *Soymovirus*, as well as of alfalfa betanucleorhabdovirus, remain unknown (Table 1) [11,12,13].

### 3.3. Sources of Legume Viruses

For legume viruses, infected seed (for the seed-borne viruses), alternative hosts, and/or viruliferous vectors (for the persistent viruses) may serve as their sources [14,29,99,114,115].

#### 3.3.1. Infected Seeds

An estimated 50% of the legume viruses are seed-borne; and most of them (the non-persistently transmitted ones) are found in the cotyledon/embryo (referred to as true seed transmission), but some (also transmitted by contact) could be carried on the seed coat, as a surface contaminant [116]. The seed transmission rate depends on the virus species and strain, host plant species and cultivar, stage of plant at which infection occurs, and environmental conditions (especially temperature). However, in some legume viruses, extremely high (up to 100%) seed transmission rates could be observed. Seed transmission is considered a very effective method of virus introduction into a crop, and an efficient survival and dispersing mechanism. Seed-borne infections result in acute symptoms and serve as randomly distributed foci of infection throughout the crop which are used for further dispersal of the virus by insect vectors. Subsequently, the onset of vector activity and population growth in the field affect the secondary virus spread and crop losses. Infected seeds might represent a mechanism of survival for many viruses over the summer months and the vehicle for long-distance spread of the non-persistent legume viruses [107,114,116,117].

A non-exhaustive list of the seed transmitted legume viruses include the following: (i) non-persistently transmitted by aphids, belonging to the genera *Alfamovirus*, *Cucumovirus*, *Fabavirus Macluravirus* and *Potyvirus*; (ii) persistently-transmitted by aphids of the genus *Enamovirus*; (iii) transmitted by aphids or whiteflies in *Carlavirus* genus; (iv) vectored by nematodes from the *Tobravirus*, and *Nepovirus* genus including SLRSV; (v) transmitted by pollen of the *Ilarvirus* genus; and (vi) transmitted by plasmodiophorids and belong to the *Pecluvirus* and *Pomovirus* genera. Viruses in the *Potexvirus* and *Tobamovirus* genera (transmitted by contact) and possibly in the *Comovirus* and *Sobemovirus* (beetle- and contact-transmitted) are carried by seeds as contaminants [10,13].

#### 3.3.2. Alternative Legume and Non-Legume Hosts

For the few legume viruses with a broad host range, e.g., CMV that infects at least 1060 species in 100 families [118], multiple virus sources (including non-legume hosts and weeds) may be available. Nonetheless, most legume viruses, especially the persistently transmitted ones, have a restricted host range, often limited to the Fabaceae family. Therefore, legume crops and particularly perennial pastures such as alfalfa, and subterranean or white clover, may serve as virus sources in between cultivation seasons of annual crops. These pastures, often sown with uncertified seeds, may create a large and continuous reservoir of the seed-transmitted legume viruses [14,29]. On the other hand, perennial pastures form a herbaceous ‘green bridge’ for legume viruses that would otherwise be unable to persist (survive) during dry summer conditions. They may also serve as overwintering hosts for the aphid vectors of the persistently transmitted legume viruses. Often overcrowded aphid vector populations on the perennial pastures are forced to disperse to the other legumes. Another cause of this movement could be multiple harvests [14]. Successive legume cultivation in the same area may also facilitate virus spread and perpetuation in between seasons [114].

#### 3.3.3. Viruliferous Vectors

The persistently transmitted viruses can be also perpetuated in the absence of susceptible hosts in their viruliferous vectors via transovarial (vertical) transmission from infected females to their progeny. Surviving, over winter or summer, virouliferous vectors on stems of perennial legumes may serve also as source of the virus [114].

### 3.4. Epidemiology of the Insect-Transmitted Legume Viruses

Epidemics of insect-transmitted viruses start with virus introduction from external virus sources (primary spread), which is followed by further spread among field plants (secondary spread). The vector species involved and the abundance and proximity of virus sources are major components in virus epidemiology. The necessary feeding time for virus acquisition and transmission shapes the vector specificity (crop colonizing or non-colonizing) and the plethora of possible vectors. Likewise, virus retention time in the insect vector determines the vicinity of the virus sources for further virus spread. For the non-persistently transmitted viruses, which are mostly transmitted by transient/migratory aphid species, the most important factor affecting the spread is the proximity to substantial virus reservoirs (due to the short retention time of the virus in the vector). On the other hand, semi- and persistently transmitted viruses might originate from distant virus sources due to the increased retention time in the insect-vectors. These viruses are introduced mostly in random distribution, by only colonizing species, and their spread is mainly affected by the vector population built up and activity in the crop [107,108,114,119].

## 4. Control Approaches for Insect-Transmitted Viruses of Legumes

Plant viruses cause systemic infections and are capable of destructive epidemics, especially those that are transmitted by insects. In the absence of antiviral substances, only integrated management approaches which combine various measures may efficiently control the virus spread. Such approaches aim to prevent virus introduction into and further spread within the crop by minimizing virus and vector reservoir(s), and reduction of the size of vector population and its movement onto the crop. Notwithstanding, in order to obtain the optimal combination of such measures, knowledge of the identity of the viruses that infect legume crops in a specific area and the epidemiological aspects of their spread is of major importance [114,120,121]. A detailed consideration of the many measures and practices which could be employed in the integrated control of legume viruses is beyond the scope of this review. Nevertheless, here we outline some of the main control approaches for the insect-transmitted legume viruses and further details can be found in other comprehensive reviews on the subject [2,5,6,14,29,99,115,122,123].

### 4.1. Elimination of Virus and Vector Sources

The use of virus-free or virus-tested seed is the main measure employed for the control of legume viruses. The use of virus tested, i.e., seeds with infection rates below accepted threshold values, is of primary importance especially for local landraces conserved in situ by farmers. Removal of volunteering legume plants or other virus and vector sources, such as weeds within or in close proximity of the crop, should be performed before sowing, while infected crop legumes should be removed (roguing) early in the season before the onset of the activity of the insect vectors. Crop rotation with cereals may also reduce virus and vector sources originating from volunteer legumes.

### 4.2. Avoiding of Virus and Vector Sources via Special Isolation

Other potentially infected crops (early season legumes or other alternative host species) and especially perennial pastures (i.e., lucerne) that may host legume viruses and aphid vectors should be avoided through spatial isolation. The effective radius for this isolation highly depends on the virus transmission mode; for the non-persistently transmitted viruses, the sources are in close proximity to the crop, while semi- and persistently transmitted viruses may also originate from distant sources, sometimes difficult to avoid.

### 4.3. Host Plant Resistance to Viral Diseases

The use of virus-resistant or -tolerant genotypes to avoid or alleviate crop losses is considered the most economical, practical, and environmentally-friendly approach. However, lack of resistant cultivars is one of the major limitations of the approach and screening of legume germplasm for resistance and its integration in elite varieties is sometimes a challenge.

### 4.4. Avoidance of Insect Vectors via Changes in Cultural Practices

Avoidance of infection is a strategy that employs several cultural practices in order to prevent incoming vectors to feed on the legume crop and they are based on the knowledge of the insect’s behavior. Some insects tend to alight preferentially or to plants surrounded by bare soil. Therefore, when establishing the crop, planting in large compact blocks of uniform age decrease the proportion of plants in the vulnerable peripheral areas. Sowing a non-host of the virus around the crop (border planting), may also delay the entry of migrating aphid into the crop and decrease the load of non-persistent viruses due to their short retention time and the probing activity performed on the non-hosts. The spread of persistent viruses may be also decreased in case the viruliferous insects colonize the non-host border and, therefore, only their non-viruliferous progenies migrate to the crop if the colonies get overcrowded.

Likewise, increasing plant density (obtained with narrow row spacing, high seeding rate) and early canopy development resulting in early bare soil cover is reducing the landing rate of incoming viruliferous vectors on young plants. Close spacing proved to decrease the proportion of infected plants within a stand. Shaded, virus-infected plants are also less vigorous and, therefore, they are not available for secondary virus spread. Groundcover or mulches may have the same effect in decreasing vector landing and exposure of young plants. Early planting is another measure that may promote early growth and the most vulnerable young plants will not be exposed to peak vector populations.

### 4.5. Chemical Control of Insect Vectors

Insecticide applications that aim to reduce vector populations may sometimes play only a limited role in plant virus control. Their efficacy highly depends on their mode of action, the mechanisms of transmission of the targeted viruses, and the type of virus spread in the field (primary or secondary) [124]. Insecticides may be successful in reducing virus spread and the final incidence of semi-persistently and persistently-transmitted viruses. These viruses are mainly spread by vectors that colonize the crop and need sufficient feeding time for acquisition and transmission that also ensures exposure to lethal insecticide doses. Compared to foliar insecticides, seed treatment may confer higher protection at the earlier stages of plant development. However, a sufficient reduction in vector population may still not result in a comparable decrease in virus spread, as infestation by a single viruliferous insect may initiate infection. On the other hand, for the non-persistently transmitted viruses, which are acquired and transmitted in brief probes, and mainly by transient aphid species that do not colonize the crop being sprayed, insecticides usually fail to control their spread. Only some pyrethroids or insect behavior-modifying chemicals may still be effective due to their rapid breakdown or anti-feedant activity. Yet, although the (secondary) spread of these viruses are not efficiently controlled in the field by the use of insecticide targeting the crop, the control of primary spread (introduction to the field) is still possible when insecticides are applied on virus and vectors sources, such as infected weeds outside the target crop (preferably before planting) or to any barrier crops. New approaches using substances that trigger plant defensive mechanisms in combination with insecticides may be promising in the control of legume viruses (e.g., [125]).

## 5. Conclusions

Legumes have gained an increasing importance in human health and sustainable agriculture in recent times. Plant viruses are considered a major constraint for legume production in many areas of the world. In the absence of efficient chemicals to treat virus-infected plants, the identification of the causal virus is of major importance in order to employ the most efficient control measures. Cutting-edge technologies explore the virome of legume species in order to identify the causal agents of major diseases, thus resulting in the discovery of new viruses. This changes our knowledge and perception of the virus status and challenges virus systematics, which should be continuously updated. However, it is the first step toward understanding the epidemiology of plant virus diseases and limiting their spread in order to ensure our future food and feed security.

## Figures and Tables

**Table 1:** The family-level classification of the legume-infecting viruses (according to the International Committee on Taxonomy of Viruses, ICTV Master Species List 2020.v1, [10]), hosts and transmission.

Family	Genus	Virus Species	Acronym	Hosts ^a^	Transmission ^bc^
*Alphaflexiviridae*					
	*Allexivirus*	Alfalfa virus S	AVS	A	Mites (SP)
	*Potexvirus*	Clover yellow mosaic virus	ClYMV	Tr	Contact, S
		White clover mosaic virus	WClMV	A, P, Tr	
		Cactus virus X	CVX	A	
*Betaflexiviridae*					
	*Carlavirus*	Cowpea mild mottle virus	CPMMV	A, B, C, FB, Gr, P	Whiteflies (NP), S
		Pea streak virus	PeSV	A, Cp, L, P	Aphids (NP), Pollen, S
		Red clover vein mosaic virus	RCVMV	A, B, Cp, FB, L, P, Tr	
*Bromoviridae*					
	*Alfamovirus*	Alfalfa mosaic virus	AMV	A, B, C, Cp, FB, L, P, Tr	Aphids (NP), S
	*Bromovirus*	Broad bean mottle virus	BBMV	B, Cp, FB, L, P	Beetles (SP), S, C
		Cowpea chlorotic mottle virus	CCMV	B, C, Gr	
	*Cucumovirus*	Cucumber mosaic virus	CMV	A, B, C, Cp, FB, Gr, L, M, P, Tr	Aphids (NP), S
		Peanut stunt virus	PSV	A, B, C, FB, Gr, L, P, Tr	
	*Ilarvirus*	Tobacco streak virus	TSV	A, B, Gr, FB, C, Cp	Pollen (thrips), S
*Caulimoviridae*					
	*Soymovirus*	Peanut chlorotic streak virus	PCSV	Gr	Uknown
*Closteroviridae*					
	*Crinivirus*	Bean yellow disorder virus	BYDV	B	Whiteflies (SP)
		Cucurbit yellow stunting disorder virus	CYSDV	A, B	
		Lettuce chlorosis virus	LCV	B	
		Tomato chlorosis virus	ToCV	C, FB	
*Geminiviridae*					
	*Becurtovirus*	Beet curly top Iran virus	BCTIV	B, C	Leafhoppers (C)
		Spinach curly top Arizona virus	SCTV	B, C	
	*Begomovirus*	Bean calico mosaic virus	BCaMV	B, C	Whiteflies (C)
		Bean chlorosis virus	BClV	B	
		Bean dwarf mosaic virus	BDMV	B	
		Bean golden mosaic virus	BGMV	B	
		Bean golden yellow mosaic virus	BGYMV	B, Gr	
		Bean leaf crumple virus	BLCrV	B	
		Bean white chlorosis mosaic virus	BWCMV	B	
		Bean yellow mosaic Mexico virus	BYMMxV	B	
		Jatropha mosaic virus	JMV	B	
		Cucurbit leaf crumple virus	CuLCrV	B	
		Common bean mottle virus	CBMoV	B	
		Common bean severe mosaic virus	CBSMV	B	
		Cotton leaf crumple virus	CLCrV	B	
		Cowpea bright yellow mosaic virus	CPGMV	C	
		Cowpea golden mosaic virus	CGMD	C	
		French bean leaf curl virus	FbLCV	B	
		Horsegram yellow mosaic virus	HgYMV	B, Cp	
		Macroptilium mosaic Puerto Rico virus	MacMPRV	B	
		Macroptilium yellow mosaic Florida virus	MacYMFV	B	
		Mungbean yellow mosaic India virus	MYMIV	B, C, Cp, L	
		Mungbean yellow mosaic virus	MYMV	B, Cp, Gr	
		Pea leaf distortion virus	PeLDV	P	
		Pepper leafroll virus	PLRV	B, Cp, L	
		Rhynchosia yellow mosaic virus	ShYMV	B	
		Sida golden mosaic virus	SiGMV	B	
		Sida micrantha mosaic virus	SiMMV	B	
		Tobacco curly shoot virus	TbCSV	B	
		Tobacco leaf curl Cuba virus	TbLCCuV	B	
		Tomato leaf curl Gujarat virus	ToLCGuV	B	
		Tomato leaf curl Joydebpur virus	ToLCJoV	B	
		Tomato leaf curl Palampur virus	ToLCPaIV	B	
		Tomato severe rugose virus	ToSRV	B	
		Tomato yellow leaf curl virus	TYLCV	B, C	
		Tomato yellow leaf curl China virus	TYLCCNV	B	
		Tomato yellow spot virus	ToYSV	B	
	*Capulavirus*	Alfalfa leaf curl virus	ALCV	A	Aphids (C)
		French bean severe leaf curl virus	FbSLCV	B	
	*Curtovirus*	Beet curly top virus		B, P	Leafhoppers (C)
	*Mastrevirus*	Chickpea chlorotic dwarf virus	CpCDV	Cp, B, FB, L	Leafhoppers (C)
		Chickpea chlorosis Australia virus	CpCAV	A, B, Cp	
		Chickpea chlorosis virus	CpCV	Cp	
		Chickpea redleaf virus	CpRLV	B, Cp	
		Chickpea yellow dwarf virus	CpYDV	B, Cp	
		Chickpea yellows virus	CpYV	B, Cp	
		Tobacco yellow dwarf virus	TYDV	A, B, Cp	
*Luteoviridae*					
	*Enamovirus*	Alfalfa enamovirus 1	EAV-1	A	Aphids (C), S
		Pea enation mosaic virus-1	PEMV-1	Cp, FB, L, P	
	*Luteovirus*	Bean leafroll virus	BLRV	A, Cp, FB, L, M, P, Tr	Aphids (C)
		Soybean dwarf virus	SbDV	A, B, Cp, FB, L, M, P, Tr	
	*Polerovirus*	Beet western yellows virus	BWYV	A, Cp, FB, L, P, Tr	Aphids (C)
		Chickpea chlorotic stunt virus	CpCSV	A, Cp, FB, L, P	
		Cotton leafroll dwarf virus	CLRDV	Cp	
		Cucurbit aphid-borne yellows virus	CABYV	Cp, FB, L	
		Faba bean polerovirus 1	FBPV-1	Cp, FB	
		Potato leafroll virus	PLRV	Cp, L	
		Turnip yellows virus	TuYV	A, Cp, FB, P	
	*Unassigned*	Chickpea stunt disease associated virus	CpSDaV	Cp	Aphid(C)
		Groundnut rosette assistor virus	GRAV	Gr	
*Nanoviridae*					
	*Nanovirus*	Cycas necrotic stunt virus	CNSV	A	Aphids (C)
		Faba bean necrotic stunt virus	FBNSV	FB	
		Faba bean necrotic yellows virus	FBNYV	A, B, C, Cp, FB, L, M, P, Tr	
		Faba bean yellow leaf virus	FBYLV	FB	
		Milk vetch dwarf virus	MDV	C, FB, P	
		Pea necrotic yellow dwarf virus	PNYDV	FB, P, Tr	
		Pea yellow stunt virus	PYSV	P	
		Subterranean clover stunt virus	SCSV	A, B, FB, M, P, Tr	
*Potyviridae*					
	*Macluravirus*	Artichoke latent virus	ArLV	L	Aphids (NP), S
	*Potyvirus*	Bean yellow mosaic virus	BYMV	A, B, C, Cp, FB, Gr, P, L, M, Tr	Aphids (NP), S
		Bean common mosaic necrosis virus	BCMNV	B, C, Gr	
		Bean common mosaic virus	BCMV	A, B, C, FB, Gr, M	
		Beet mosaic virus	BtMV	Cp	
		Bidens mosaic virus	BiMV	FB, P	
		Bidens mottle virus	BiMoV	FB	
		Clover yellow vein virus	ClYVV	A, B, FB, P, Tr	
		Cowpea aphid-borne mosaic virus	CABMV	B, C, Gr	
		Lettuce mosaic virus	LMV	Cp, P	
		Papaya ringspot virus	PRSV	P	
		Passion fruit woodiness virus	PWV	Gr, P	
		Pea seed-borne mosaic virus	PSbMV	A, B, FB, Cp, L, P	
		Peanut mottle virus	PeMoV	B, C, Gr, M, P, Tr	
		Plum pox virus	PPV	Tr	
		Soybean mosaic virus	SMV	A, B, C	
		Turnip mosaic virus	TuMV	Cp, FB, P	
		Watermelon mosaic virus	WMV	M, P	
		Wisteria vein mosaic virus	WVMV	B	
		Zucchini yellow mosaic virus	ZYMV	Tr	
*Rhabdoviridae*					
	*Betanucleorhabdovirus*	Alfalfa betanucleorhabdovirus	AaNV	A	Uknown
	*Cytorhabdovirus*	Alfalfa dwarf cytorhabdovirus	ADV	A	Aphids (C-Pr)
		Lettuce necrotic yellows virus	LNYV	Cp	
*Secoviridae*					
	*Cheravirus*	Cherry rasp leaf virus	CRLV	B	Nematodes (SP)
	*Comovirus*	Broad bean stain virus	BBSV	FB, L, P	Beetles (SP), S, Contact
		Bean pod mottle virus	BPMV	B, C	
		Bean rugose mosaic virus	BRMV	B, FB	
		Broad bean true mosaic virus	BBTMV	FB, P	
		Cowpea mosaic virus	CPMV	C	
		Cowpea severe mosaic virus	CPSMV	C, B	
		Pea green mottle virus	PGMV	P	
		Pea mild mosaic virus	PMiMV	P	
		Quail pea mosaic virus	QPMV	B, C	
		Red clover mottle virus	RCMV	Tr	
	*Fabavirus*	Broad bean wilt virus 1	BBWV-1	(B, Cp, FB, L, P) ^d^	Aphids (NP), S
		Broad bean wilt virus 2	BBWV-2		
	*Nepovirus*	Arabis mosaic virus	ArMV	Tr	Nematodes (SP), S
		Artichoke yellow ringpsot virus	AYRSV	FB	
		Crimson clover latent virus	CCLV	Tr	
		Cycas necrotic stunt virus	CNSV	A	
		Lucerne Australian latent virus	LALV	A, M, Tr	
		Tobacco ringspot virus	TRSV	B, C, Cp	
		Tomato black ring virus	TBRV	B, L, Tr	
		Tomato ringspot virus	ToRSV	Tr	
	*Unassigned*	Strawberry latent ringspot virus	SLRSV	A, Tr	Nematodes, S
*Solemoviridae*					
	*Sobemovirus*	Lucerne transient streak virus	LTSV	A, Tr	Contact (beetles, aphids), S
		Southern bean mosaic virus	SBMV	B, C	
		Southern cowpea mosaic virus	SCPMV	C	
		Subterranean clover mottle virus	SCMoV	M, Tr	
*Tombusviridae*					
	*Dianthovirus*	Red clover necrotic mosaic virus	RCNMV	A, Tr	Chytrids (P), Soil
		Sweet clover necrotic mosaic virus	SCNMV	A	
	*Gammacarmovirus*	Cowpea mottle virus	CPMoV	C	Beetles, S
		Pea stem necrosis virus	PSNV	P	Chytrids (P)
	*Alphanecrovirus*	Tobacco necrosis virus A	TNV-A	B	Chytrids (P)
	*Tombusvirus*	Cymbidium ringspot virus	CymRSV	Tr	Chytrids (P), Contact, Soil
		Pelargonium leaf curl virus	PLCV	Tr	
		Moroccan pepper virus	MPV	Tr	
	*Umbravirus*	Pea enation mosaic virus 2	PEMV-2	A, Cp, FB, L, P, Tr	Aphids (C), assistor-dependent
		Groundnut rosette virus	GRV	Gr	
		Ethiopian tobacco bushy top virus	ETBTV	B	
	*Unassigned*	Bean mild mosaic virus	BMMV	B	Beetles
*Tospoviridae*					
	*Orthotospovirus*	Bean necrotic mosaic orthotospovirus	BeNMV	B	Thrips (C-Pr)
		Capsicum chlorosis orthotospovirus	CaCV	Gr	
		Groundnut bud necrosis orthotospovirus	GBNV	B, C, Gr, P	
		Groundnut chlorotic fan spot orthotospovirus	GCFSV	Gr	
		Groundnut ringspot orthotospovirus	GRSV	Gr, P	
		Groundnut yellow spot orthotospovirus	GYSV	Gr	
		Impatiens necrotic spot orthotospovirus	INSV	Gr	
		Melon yellow spot orthotospovirus	MYSV	FB	
		Tomato chlorotic spot orthotospovirus	TCSV	B, C, Gr	
		Tomato spotted wilt orthotospovirus	TSWV	B, C, Cp, FB, Gr, L, P, Tr	
*Tymoviridae*					
	*Marafivirus*	Alfalfa virus F	AVF	A	Leafhoppers (C-Pr)
	*Tymovirus*	Peanut yellow mosaic virus	PeYMV	Gr	Beetles (SP), Contact
*Virgaviridae*					
	*Pecluvirus*	Indian peanut clump virus	IPCV	Gr	Plasmodiophorids (P), S
		Peanut clump virus	PCV	Gr	
	*Pomovirus*	Broad bean necrosis virus	BBNV	FB	Plasmodiophorids (P), S
	*Tobravirus*	Pea early-browning virus	PEBV	A, B, FB, P	Nematodes (SP), S
	*Tobamovirus*	Sunn-hemp mosaic virus	SYHMV	C	Contact, S
		Tobacco mosaic virus	TMV	A, B, C, FB	
		Tomato mosaic virus	ToMV	A, B	
		Tomato mottle mosaic virus	ToMMV	Cp	

^a^ A, alfalfa; B, common bean; C, cowpea; Cp, chickpea; FB, faba bean; L, lentil; M, annual medics; P, pea; Tr, *Trifolium* spp. ^b^ Sa, Sap; Se, seeds; NP, non-persistent transmission; P, persistent transmission; SP, semi-persistent transmission; Chytrids, *Olpidium* spp; Plasmophorids, *Plasmodiophora* spp. ^c^ Transmission mode may apply to the virus genus [10,12,13] if it is not yet recorded specifically for the listed virus. ^d^ Host range recorded for BBWV.

## Data Availability

Data is included within the manuscript.
